# Comparison of Barium and Arsenic Concentrations in Well Drinking Water and in Human Body Samples and a Novel Remediation System for These Elements in Well Drinking Water

**DOI:** 10.1371/journal.pone.0066681

**Published:** 2013-06-21

**Authors:** Masashi Kato, Mayuko Y. Kumasaka, Shoko Ohnuma, Akio Furuta, Yoko Kato, Hossain U. Shekhar, Michiyo Kojima, Yasuko Koike, Nguyen Dinh Thang, Nobutaka Ohgami, Thuy Bich Ly, Xiaofang Jia, Husna Yetti, Hisao Naito, Gaku Ichihara, Ichiro Yajima

**Affiliations:** 1 Unit of Environmental Health Sciences, Department of Biomedical Sciences, College of Life and Health Sciences, Chubu University, Kasugai, Japan; 2 Voluntary Body for International Health Care in Chubu University, Kasugai, Japan; 3 Department of Occupational and Environmental Health, Nagoya University Graduate School of Medicine, 65 Tsurumai-cho, Showa-ku, Nagoya, Japan; 4 Departments of Biochemistry and Molecular Biology, University of Dhaka, Dhaka, Bangladesh; 5 Aichi Prefectural Institute of Public Health, Nagoya, Japan; 6 Department of Biochemistry and Plant Physiology, Faculty of Biology, Vietnam National University of Science, Hanoi, Vietnam; 7 Institute for Environmental Science and Technology, Hanoi University of Science and Technology, Hanoi, Vietnam; Biological Research Centre of the Hungarian Academy of Sciences, Hungary

## Abstract

Health risk for well drinking water is a worldwide problem. Our recent studies showed increased toxicity by exposure to barium alone (≤700 µg/L) and coexposure to barium (137 µg/L) and arsenic (225 µg/L). The present edition of WHO health-based guidelines for drinking water revised in 2011 has maintained the values of arsenic (10 µg/L) and barium (700 µg/L), but not elements such as manganese, iron and zinc. Nevertheless, there have been very few studies on barium in drinking water and human samples. This study showed significant correlations between levels of arsenic and barium, but not its homologous elements (magnesium, calcium and strontium), in urine, toenail and hair samples obtained from residents of Jessore, Bangladesh. Significant correlation between levels of arsenic and barium in well drinking water and levels in human urine, toenail and hair samples were also observed. Based on these results, a high-performance and low-cost adsorbent composed of a hydrotalcite-like compound for barium and arsenic was developed. The adsorbent reduced levels of barium and arsenic from well water in Bangladesh and Vietnam to <7 µg/L within 1 min. Thus, we have showed levels of arsenic and barium in humans and propose a novel remediation system.

## Introduction

Pollution of well drinking water with toxic elements is a serious public health problem throughout the world. About 130 million people worldwide suffer from drinking water containing >10 µg/L arsenic [1], [2], whereas the upper limit of arsenic for drinking water in the WHO health-based guidelines for drinking water is 10 µg/L. Bangladesh has been suggested to be one of the counties in the largest number of patients with arsenicosis caused by arsenic-polluted well drinking water [3]. In fact, more than 25 million patients with arsenicosis have been reported in Bangladesh [4], [5]. More than 10 million patients with arsenicosis in Vietnam have also been reported [6]. Furthermore, epidemiological studies have revealed increases in arsenic-mediated carcinomas, skin diseases, neurologic diseases and cardiovascular diseases [2], [7]–[9]. Experimental studies have also provided evidence that arsenic promotes these diseases [10]–[12]. There have also been several studies on arsenic levels in well drinking water samples and human samples such as urine, nails and hair [4], [13], [14]. A recent review paper suggested that arsenic levels in urine and toenails could be a useful biomarker for assessing arsenic exposure via drinking water [1].

On the other hand, the evidence of arsenic-mediated diseases does not rule out the possibility that another element as well as arsenic in well water contributes to diseases in humans. In fact, well drinking water in Bangladesh and Vietnam has been reported to contain not only arsenic but also various elements such as magnesium, calcium, strontium, barium, iron, manganese, uranium and chromium [15]–[19]. Some elements such as manganese, iron and zinc for which values were shown in the previous edition of WHO health-based guidelines for drinking water were omitted in the current 4th edition of the guidelines revised in 2011. However, the guideline values of 700 µg/L for barium and 10 µg/L for arsenic have been maintained in the current edition. Our previous *in vitro* study also showed that a low level (343.3–686.6 µg/L) of barium alone promotes transforming activity of nontumorigenic keratinocytes, fibroblasts and melanocytes [18]. Our study further showed that a low level of barium (137.3 µg/L) promotes arsenic (224.8 µg/L)-mediated cancer toxicity [19]. Our previous *in vivo* study also showed development of hearing loss with morphological disturbance of inner ears in wild-type mice drinking water containing 700 µg/L barium for 2 weeks [20]. Taken together, these results indicated that the health risk of barium in drinking water might not be low. To our knowledge, however, there have been very few studies on levels of barium in drinking water and human urine, nail and hair samples. Moreover, high-performance and low-cost methods for remediating barium in addition to arsenic from well drinking water are limited.

Barium, magnesium, calcium and strontium are homologous elements. In this study, we compared the levels of arsenic and these 4 elements in well drinking water and human urine, nail and hair samples to determine whether the elements ingested from water affect the levels in the human body. We further proposed a novel adsorbent composed of Mg-Fe-based hydrotalcite-like compounds [Mg(II)_4_Fe(III)_2_(OH)_12_]^2+^[NO_3_xCO_3_⋅zH_2_O]^2−^ (MF-HT) for remediation of arsenic and barium in well drinking water.

## Materials and Methods

### Ethics Statement

All experiments were performed in Chubu University. Sampling in Bangladesh and Vietnam was ethically approved (approval no. 20008–10) by the committee of Chubu University in Japan following the regulations of the Japanese government. Participants and owners of wells in Bangladesh and Vietnam provided written informed consent to participate in this study.

### Measurements of Elements in Well Drinking Water and Human Samples

Well drinking water samples (n = 30) and human urine, toenail and hair samples (n = 30; male/female = 11/19, mean ± SD of age = 36.9±13.4) were collected in previously reported cancer-prone areas of Jessore, Bangladesh [19, 21–23). Samples (n = 5) of well water were also collected in Ha Nam Province in Vietnam where pollution of arsenic in well water has been previously reported [24]. Levels of elements in the samples were measured by the method previously described [20], [25]. In brief, human samples were placed in a 15 ml polypropylene tube in the presence of 3 ml of nitric acid (61%). The tubes were capped and incubated at 80°C for 48 hrs, followed by cooling for 1 hr to room temperature. After cooling, 3 ml of hydrogen peroxide (30%) was added to each tube, followed by incubation at 80°C for 3 hrs. After suitable dilution of the digested materials with ultrapure water, levels of elements in the samples were determined by an inductively coupled plasma-mass spectrometer (ICP-MS; 7500cx, Agilent Technologies, Inc.) with a reaction cell for absence of ArCl ion interference. Urinary total element concentration was divided by creatinine concentration to obtain a creatinine-adjusted urinary total element concentration, expressed as µg/g of creatinine [26].

### Statistical Analysis

Statistical analyses using the Mann-Whitney *U* test and Spearman’s rank correlation test were performed following the method previously described [27]. The SPSS (version 18) software package (SPSS Japan Inc.) was used for these statistical analyses. The significance level was set at p<0.05.

### Synthesis and Characterization of MF-HT

The method for producing MF-HT has been described previously [21]. The solid structure was analyzed by an X-Ray Diffractometer (XRD; Rigaku RINT2000) as previously described [21]. Less than 250 µm of particles in MF-HT was used. XRD patterns of dried powder samples of MF-HT used in this study were presented in our previous report [21].

### Experiments for Adsorption

Barium chloride (Wako Pure Chemical Industries) was used as barium. Since the range of pH in well drinking water in Bangladesh is between 6.0 and 8.0, all adsorption experiments using barium-containing solutions were performed after pH in the solutions had been adjusted to 7.0 at room temperature. An adsorption experiment using well drinking water from Bangladesh was performed at room temperature without adjusting pH. Adsorption experiments using the barium-containing solution and well water from Bangladesh and Vietnam were performed by the following method. The solutions mixed with MF-HT were shaken at 300 rpm for the indicated times. After the suspensions had been centrifuged, barium concentration in the supernatants to which 10% volume of 1 M HNO_3_ (final concentration of 0.1 M HNO_3_) had been added were measured by an ICP-MS. Langmuir isotherms were used to evaluate the equilibrium data using MF-HT as describes previously [21], [28].

## Results

### Levels of 5 Elements in Well Drinking Water and Human Samples

We first measured concentrations (mean ± SD) of magnesium, calcium, strontium and barium in addition to arsenic in well drinking water samples (n = 30) and human urine, hair and nail samples (n = 30) from residents of Jessore, Bangladesh (Table 1). The mean value of arsenic in well drinking water was >13-fold higher than the value in WHO health-based guidelines (10 µg/L) for drinking water. Although the mean concentration of barium in well drinking water was lower than the value in the guidelines, the well drinking water contained more than 100 µg/L of barium. There are no values in the guidelines for magnesium, calcium and strontium, which are control homologous elements for barium, in drinking water, though the concentrations of magnesium, calcium and strontium in well water were higher than those of arsenic and barium. Mean urinary levels of arsenic, magnesium, calcium and strontium were higher than or comparable to the mean levels in well drinking water. Urinary concentration of barium was lower than the well drinking water concentration. In addition, levels of the 4 homologous elements of barium in nail samples were lower than levels in hair samples, whereas levels of arsenic in nail samples were higher than levels in hair samples.

**Table 1:** Levels of 5 elements in well water and human samples obtained in Bangladesh.

	As	Mg	Ca	Sr	Ba
**Well water** (µg/L)	133±153	23,228±4,158	65,766±14084	224±53	103±49
**Urine** (µg/g of creatinine)	124±197	84,860±51,710	128,018±109,489	260±194	27±68
**Nail** (µg/kg)	2,667±2887	47,347±38294	710,184±480,686	768±686	1,873±1,192
**Hair** (µg/kg)	1,823±1,844	304,819±159,273	1,952,499±969,163	6,413±2,693	4,457±2,810

Concentrations (means ± SD, n = 30) of arsenic, magnesium, calcium, strontium and barium in well drinking water and human urine, toenail and hair samples obtained in Bangladesh are shown.

### Correlations between Levels of 5 Elements in Well Drinking Water Samples and Levels in Human Samples

To examine whether ingested elements from drinking water affect levels of the elements in the human body, we next investigated correlations between levels of arsenic, magnesium, calcium, strontium and barium in well drinking water samples and levels in urine, nail and hair samples (Table 2). There were significant correlations between levels of arsenic in well drinking water samples and levels in urine (r = 0.68), nail (r = 0.73) and hair (r = 0.74) samples. Significant correlations were also found between levels of barium in well drinking water samples and levels in human urine (r = 0.41), nail (r = 0.43) and hair (r = 0.59) samples. However, no correlations were found between levels of magnesium, calcium and strontium in well drinking water samples and levels in the human samples except for a significant correlation (r = 0.54) between levels of calcium in well drinking water samples and hair samples.

**Table 2:** Correlations between well water and human samples for each element.

	Well Water				
	As	Mg	Ca	Sr	Ba
**Urine**	0.68**	0.24	−0.10	0.22	0.41*
**Nail**	0.73**	−0.27	−0.03	−0.38	0.43*
**Hair**	0.74**	−0.17	0.54**	0.15	0.59**

Spearman’s rank correlation coefficient (n = 30) for correlation between drinking well water and human urine, toenail and hair samples in Bangladesh in each element (arsenic, magnesium, calcium, strontium and barium) are shown. * and **, Statistically significant correlation (*, p<0.05; **, p<0.01).

### Correlation between Levels of Arsenic and Levels of Barium Homologous Elements in Well Drinking Water Samples and Human Samples

We next investigated correlations between levels of arsenic and levels of barium homologous elements (magnesium, calcium, strontium and barium) in well drinking water and human urine, nail and hair samples (Table 3). Significant correlations were found between levels of arsenic and levels of the 4 elements in well drinking water samples, while the correlation between arsenic and strontium was negative (r = −0.66). There were significant correlations between levels of arsenic and levels of barium in urine (r = 0.46), nail (r = 0.53) and hair (r = 0.56) samples despite the fact that there was no significant correlation between levels of arsenic and levels of the other 3 elements (magnesium, calcium and strontium) in human samples.

**Table 3:** Correlations between arsenic and other elements in well water and human samples.

		Mg	Ca	Sr	Ba
**As**	**Well water**	0.74**	0.74**	−0.66**	0.87**
	**Urine**	0.34	0.11	0.18	0.46*
	**Nail**	0.25	0.06	0.22	0.53**
	**Hair**	0.05	0.14	−0.14	0.56**

Spearman’s rank correlation coefficients (n = 30) for correlation between arsenic and other elements (magnesium, calcium, strontium and barium) in well drinking water and human urine, toenail and hair samples obtained in Bangladesh are shown. * and **, Statistically significant correlation (*, p<0.05; **, p<0.01).

### Kinetics for Barium Adsorption in MF-HT

**Figure 1:**
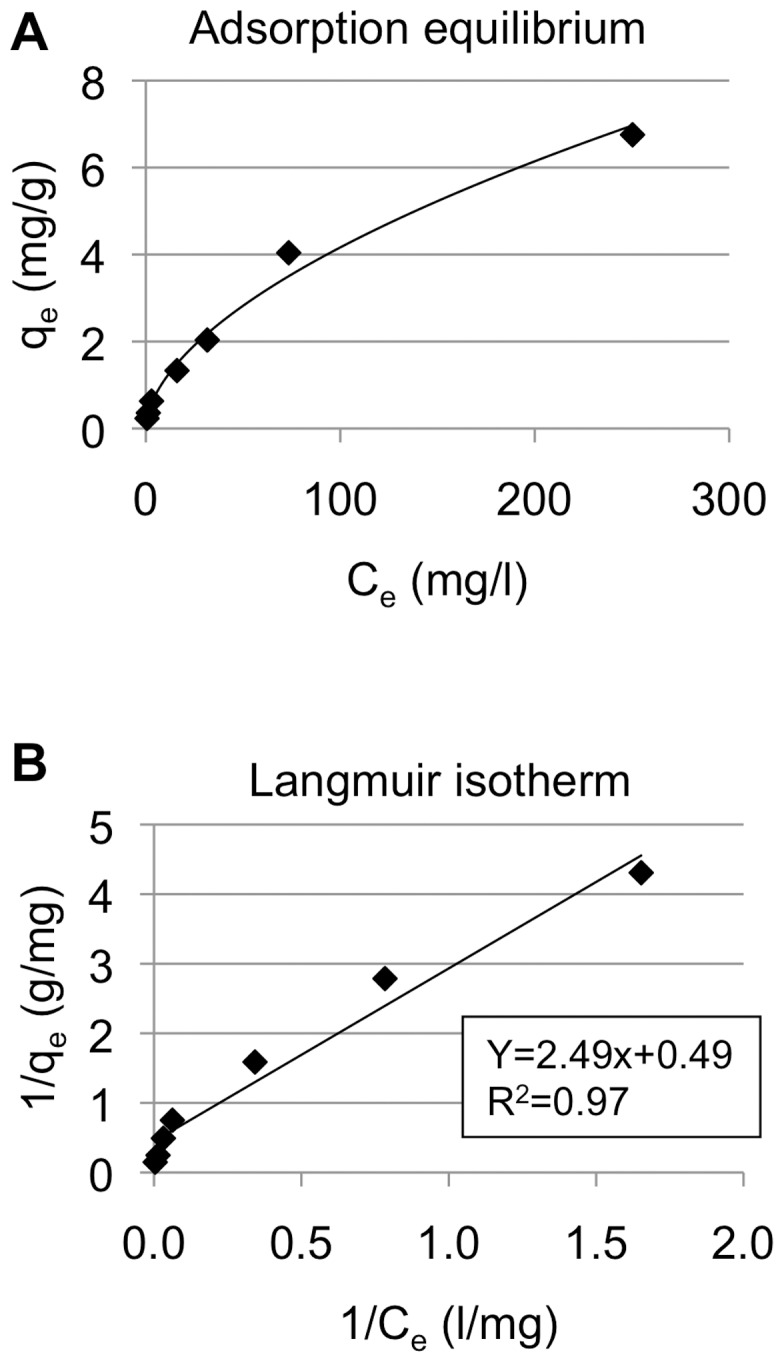
Quality of MF-HT. Equilibrium data (a) and Langmuir isotherm (b) for barium adsorption in MF-HT are presented.

Based on results of the present study for concentrations of arsenic and barium and previous results showing toxicity of arsenic and barium [10], [18]–[20], we tried to establish a method for remediation for both arsenic and barium from well drinking water. Since our previous study already provided evidence that MF-HT could adsorb arsenic in water [21], we first determined the adsorption ability of MF-HT for barium in water. Adsorption kinetics and isotherms were examined in batch experiments (Fig. 1). Langmuir isotherms were used to analyze the equilibrium adsorption results for barium in MF-HT. Our equilibrium data for barium adsorption in MF-HT (Fig. 1a) and the Langmuir isotherm for barium adsorption in MF-HT (R^2^>0.95; Fig. 1b) show barium adsorption by MF-HT.

### Amount of Barium Adsorption in MF-HT

Since we obtained evidence of MF-HT-mediated barium adsorption (Fig. 1), we measured the amount of adsorption in MF-HT. After the indicated percents of weight of MF-HT (weight of MF-HT/weight of solution) had been added to solutions with 1,000 µg/L barium and sequentially shaken for 60 min, barium concentrations in the solutions were measured (Table 4). Our results showed that 0.010% weight of MF-HT could adsorb 1,000 µg/L barium in the solutions to less than 700 µg/L, the value of WHO health-based guidelines for drinking water.

**Table 4:** Barium absorption in the indicated percent of weight of MF-HT.

Percent of MF-HT (wt/wt)	Barium concentration Mean ± SD (µg/L) (n = 3)
0.001%	962.30±23.83
0.010%	621.40±19.77
0.100%	270.63±35.40
1.000%	71.80±8.52

After the indicated percentages of weight of MF-HT (percent of MF-HT) had been suspended in solutions containing 1,000 µg/L barium, the MF-HT-suspended solutions were shaken at 300 rpm for 1 hour. Barium concentrations (means ± SD) in the supernatants after the solutions had been centrifuged are presented.

### Adsorption Time for Barium in MF-HT

We next examined MF-HT-mediated adsorption time for 1,000 µg/L barium solutions. The concentration of barium in the solution treated with 1% weight of MF-HT was reduced from 1,000 µg/L to 60–70 µg/L within 15 sec (Table 5). Since adsorption for a short time is important for application of MF-HT to drinking water, this result encourages practical use of MF-HT for barium-polluted drinking water.

**Table 5:** Barium absorption time in 1% of weight of MF-HT.

Time (sec)	Barium concentration Mean ± SD (µg/L) (n = 3)
15	62.0±1.9
30	67.9±4.1
60	71.8±1.9

After 1% of weight of MF-HT had been suspended in solutions containing 1,000 µg/L barium, the solutions were shaken at 300 rpm for the indicated time. Barium concentrations (means ± SD) in the supernatants after the solutions had been centrifuged are presented.

### Adsorption of Barium in Well Water by MF-HT

**Figure 2:**
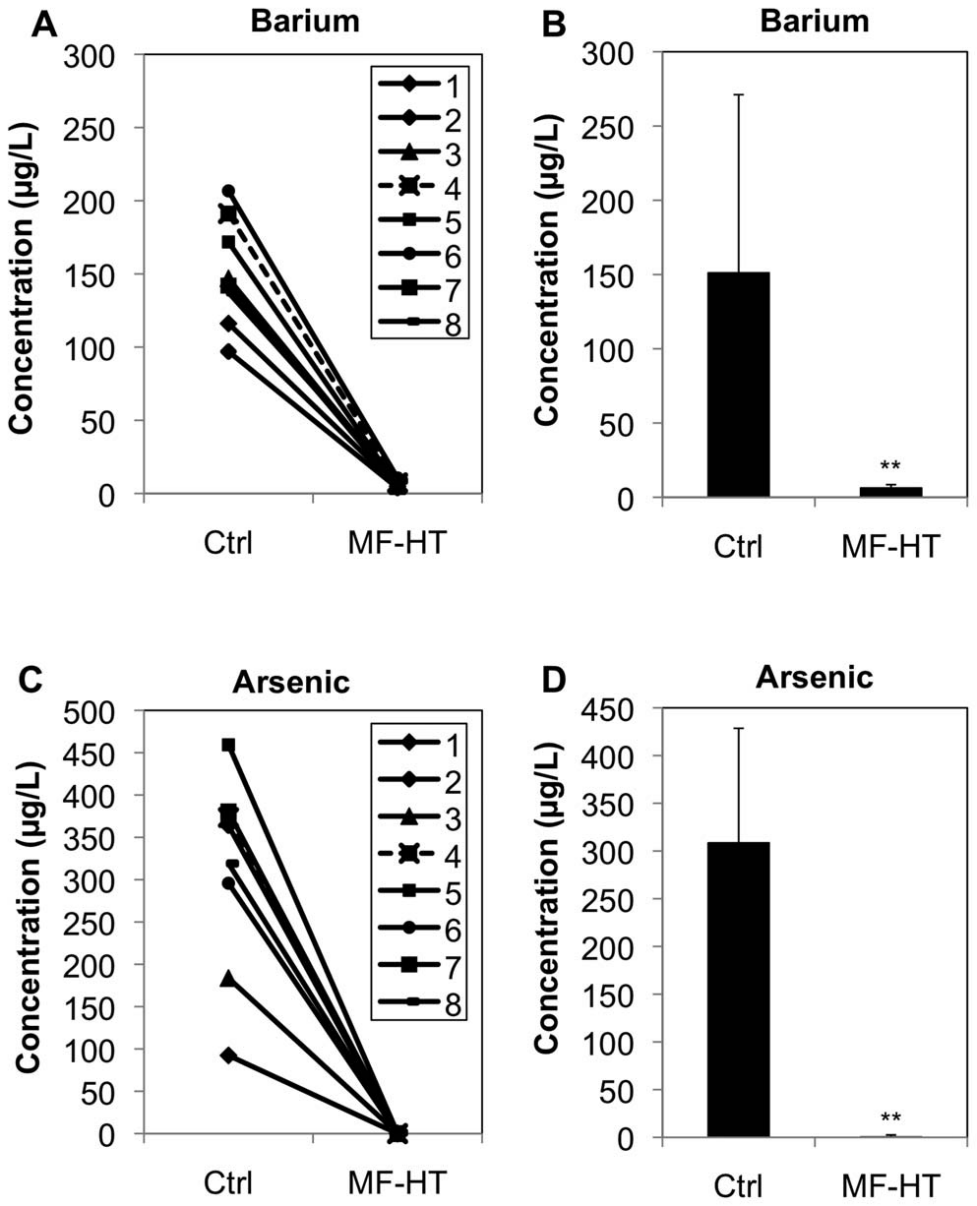
MF-HT-mediated adsorption of barium and arsenic from well water in Bangladesh. Well water samples (n = 8) from Jessore, Bangladesh with barium (range: 97.0–206.7 µg/L) not exceeding the value in WHO health-based guidelines and arsenic (range: 92.3–459.3 µg/L) exceeding the value in the guidelines were prepared. After 1% weight of MF-HT had been suspended in the well water samples, the MF-HT-suspended solutions were shaken at 300 rpm for 1 min. After the solutions had been centrifuged, barium (a and b) and arsenic (c and d) concentrations in the MF-HT-treated supernatants (MF-HT) were compared with those in untreated control water samples (Ctrl). **, Statistically different (p<0.01) from the control by the Mann-Whitney *U* test.

**Figure 3:**
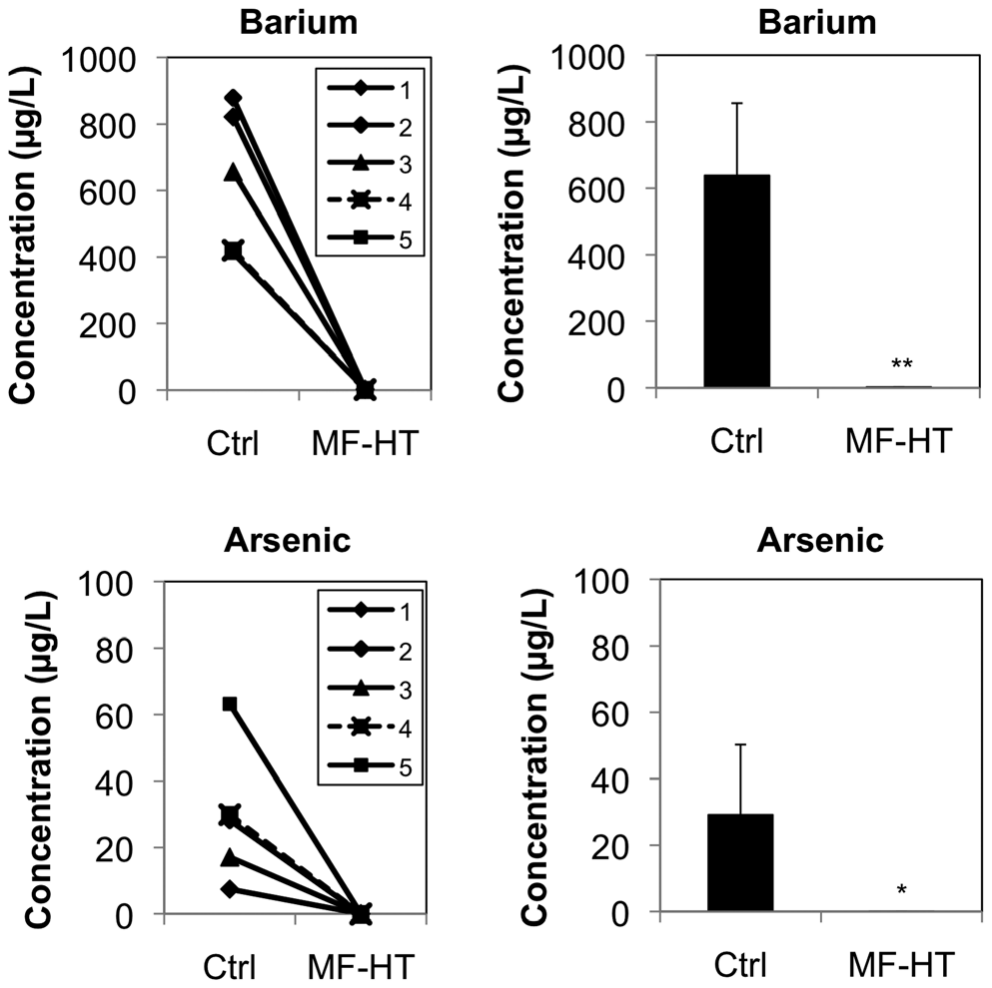
MF-HT-mediated adsorption of barium and arsenic from well water in Vietnam. Well water samples (n = 5) from Ha Nam, Vietnam with barium (range: 412.8–879.6 µg/L) and arsenic (range: 7.4–63.2 µg/L) were prepared. After 1% weight of MF-HT had been suspended in the well water samples, the MF-HT-suspended solutions were shaken at 300 rpm for 1 min. After the solutions had been centrifuged, barium (a and b) and arsenic (c and d) concentrations in the MF-HT-treated supernatants (MF-HT) were compared with those in untreated control water samples (Ctrl). * and **, Statistically different (*, p<0.05; **, p<0.01) from the control by the Mann-Whitney *U* test.

We finally examined whether MF-HT is able to adsorb barium from well water. Adsorption experiments were performed using well drinking water samples (n = 8) containing barium (mean = 151.1 µg/L) and arsenic (mean = 298.2 µg/L) that were obtained in Jessore, Bangladesh (Fig. 2). Mean concentrations of barium and arsenic in the well water samples treated with 1% weight of MF-HT for 1 min were decreased to 6.3 µg/L and 0.5 µg/L, respectively. Since none of the well water samples obtained in Bangladesh contained barium exceeding the value in WHO health-based guidelines (700 µg/L), we further performed adsorption experiments using well water samples from Vietnam (n = 5), in which 3 of the 5 samples and 4 of the 5 samples exceeded the guideline values of barium and arsenic, respectively (Fig. 3). Both concentrations of barium and arsenic in the well water samples treated with 1% weight of MF-HT for 1 min were decreased to <7 µg/L. The results indicated that MF-HT can be used as a depurative for well water polluted by barium as well as arsenic.

## Discussion

We demonstrated concentrations of magnesium, calcium, strontium and barium in addition to arsenic in well drinking water and human urine, hair and nail samples obtained in Bangladesh. Our comparative analysis of concentrations of barium showed that the amount of barium egested in urine is small compared with the amount of arsenic, magnesium, calcium and strontium. Moreover, barium homologous elements tend to accumulate in hair rather than nails, while arsenic has a tendency to accumulate in nails rather than hair in humans. Our results for barium levels in urine and nails suggest that levels of barium in the samples may not be suitable for biomarkers to assess exposure. We also demonstrated significant correlations between levels of arsenic and barium in well drinking water and levels in urine, nail and hair samples. Our results for arsenic agree with previous results showing correlations between levels of arsenic in well drinking water samples and levels in urine (r = 0.774), nail (r = 0.719) and hair (r = 0.773) samples obtained in West Bengal, India and Bangladesh (n = 31–59) [13]. In addition, our results for arsenic concentrations in well drinking water and human urine, nail and hair samples are comparable to results of previous studies in Bangladesh [4], [13], [14]. We next demonstrated significant correlations between levels of arsenic and levels of barium in urine, nail and hair samples from residents of Bangladesh, though there was no correlation between levels of arsenic and levels of magnesium, calcium or strontium in the human samples. Taken together with results of our previous study showing modification of arsenic toxicity by coexposure to barium [19], our results suggest effects of barium on the pathogenesis of arsenic-mediated diseases.

Our previous studies showed toxicity of barium and/or arsenic at low concentrations [18]–[20]. More importantly, the values of arsenic (10 µg/L) and barium (700 µg/L) remain in the current edition of WHO health-based guidelines for drinking water. Therefore, we proposed a novel method to adsorb barium in addition to arsenic from drinking water. After barium adsorption through MF-HT was verified by an adsorption experiment using Langmuir isotherm, we calculated that about 2 L of 1,000 µg/L barium-polluted water could be theoretically adsorbed to less than the levels in the guidelines by 1 g MF-HT. Since iron is cheaper than aluminum, MF-HT consisting of magnesium and iron would be cheaper than the hydrotalcite consisting of magnesium and aluminum, which can be produced at a cost of US$ 700/ton [29]. Less than 0.7 cent per day may be sufficient to depurate 20 L of drinking water for consumption by a family per day since our results showed that 20 L of well water maximally polluted by barium and arsenic in Vietnam could be theoretically depurated by 10 g MF-HT. Moreover, production would be possible in developing countries because the method for producing MF-HT is not difficult. However, we should consider safe disposal of MF-HT containing barium and arsenic, because disposal of the adsorbent might be a secondary threat for environmental pollution and human health. Development of a disposal system for the adsorbent is needed for practical use.

Barium concentration initially set as 1,000 µg/L was decreased to 60–70 µg/L by treatment with 1% weight of MF-HT within 1 min in the solutions used in our study. On the other hand, initial mean concentration of 638.3 µg/L barium was decreased to <7 µg/L by the same treatment with MF-HT in well water. These results indicated that the adsorption ability of MF-HT for barium in well water is greater than that in an artificial barium solution. We have no evidence to explain the reason for the different adsorption abilities of MF-HT in barium solution and barium-containing well water. Since there are various substances in well water, including carbonate ion, that affect MF-HT-mediated adsorption, the adsorption ability of MF-HT may be enhanced.

In conclusion, we investigated levels of barium and arsenic and analyzed their correlation in drinking water and the human body. According to the WHO health-based guidelines, we then proposed hydrotalcite-like compound MF-HT as a novel candidate for sustainable depuration of barium and arsenic from well drinking water. Since MF-HT has high performance and can be produced easily at low cost, it may be useful in countries in which drinking water is polluted with arsenic and barium.
